# Physical activity reduces the risk of incident type 2 diabetes in general and in abdominally lean and obese men and women: the EPIC–InterAct Study

**DOI:** 10.1007/s00125-012-2532-2

**Published:** 2012-04-21

**Authors:** The InterAct Consortium

**Affiliations:** 1 The InterAct Project, http://www.inter-act.eu; 2 c/o U. Ekelund MRC Epidemiology Unit, Institute of Metabolic Science, Addenbrooke’s Hospital, Box 285, Cambridge, CB2 0QQ UK

**Keywords:** Abdominal obesity, Case–cohort study, Incident diabetes, Obesity, Physical activity

## Abstract

**Aims/hypothesis:**

We examined the independent and combined associations of physical activity and obesity with incident type 2 diabetes in men and women.

**Methods:**

The InterAct case–cohort study consists of 12,403 incident type 2 diabetes cases and a randomly selected subcohort of 16,154 individuals, drawn from a total cohort of 340,234 participants with 3.99 million person-years of follow-up. Physical activity was assessed by a four-category index. Obesity was measured by BMI and waist circumference (WC). Associations between physical activity, obesity and case-ascertained incident type 2 diabetes were analysed by Cox regression after adjusting for educational level, smoking status, alcohol consumption and energy intake. In combined analyses, individuals were stratified according to physical activity level, BMI and WC.

**Results:**

A one-category difference in physical activity (equivalent to approximately 460 and 365 kJ/day in men and women, respectively) was independently associated with a 13% (HR 0.87, 95% CI 0.80, 0.94) and 7% (HR 0.93, 95% CI 0.89, 0.98) relative reduction in the risk of type 2 diabetes in men and women, respectively. Lower levels of physical activity were associated with an increased risk of diabetes across all strata of BMI. Comparing inactive with active individuals, the HRs were 1.44 (95% CI 1.11, 1.87) and 1.38 (95% CI 1.17, 1.62) in abdominally lean and obese inactive men, respectively, and 1.57 (95% CI 1.19, 2.07) and 1.19 (95% CI 1.01, 1.39) in abdominally lean and obese inactive women, respectively.

**Conclusions/interpretation:**

Physical activity is associated with a reduction in the risk of developing type 2 diabetes across BMI categories in men and women, as well as in abdominally lean and obese men and women.

**Electronic supplementary material:**

The online version of this article (doi:10.1007/s00125-012-2532-2) contains peer-reviewed but unedited supplementary material, which is available to authorised users.

## Introduction

Obesity and low levels of physical activity are among the most important modifiable risk factors for type 2 diabetes [1–6]. Moreover, previous observational studies have suggested that higher levels of physical activity are associated with lower risk of diabetes independently of obesity [3–6]. However, few previous studies have been sufficiently large to examine the combined and stratified associations of physical activity and adiposity with incident diabetes [7–10]. Where studies have been undertaken, they have been restricted to homogeneous samples of men [8] or women [7, 10], or have dichotomised the population into only two activity groups (active versus inactive) [7, 8] or into obese versus non-obese categories [9]. Results from these studies vary in their conclusions about whether physical activity protects against the risk of type 2 diabetes across subgroups of measures of adiposity. Given the high prevalence of overweight or obesity worldwide, it is important to clarify this uncertainty, thus helping provide clear messages for primary prevention in a context where general and abdominal adiposity may be differentially and independently associated with risk of type 2 diabetes and premature death [11, 12].

Meta-analysis of existing published cohort studies, which has been attempted in this context to examine the main association of physical activity [13], is unlikely to resolve the above uncertainty because of the marked heterogeneity in the methods used to define physical activity in different cohorts. We, therefore, examined the independent and stratified associations of overall physical activity with BMI and waist circumference (WC) as part of the European Prospective Investigation into Cancer and Nutrition (EPIC)**–**InterAct Study. The EPIC–InterAct Study is a case–cohort study nested within a large pan-European study that involves male and female participants from diverse social and occupational backgrounds, and used a standardised and validated assessment of physical activity at baseline.

## Methods

### Study design

The participants, methods, study design and measurements have been described previously [14]. Briefly, the EPIC**–**InterAct project aims to examine: (1) gene × lifestyle interactions in relation to the risk of developing type 2 diabetes; and (2) how knowledge about such interactions may be translated into preventative action (www.inter-act.eu, accessed 30 September 2011). A case–cohort study of incident type 2 diabetes (InterAct Study) was established on the basis of incident type 2 diabetes cases occurring in the EPIC cohorts between 1991 and 2007 at 26 centres from eight (Denmark, France, Germany, Italy, Spain, Sweden, UK, the Netherlands) of ten EPIC countries. Due to minor differences in measurements of anthropometry and physical activity between centres in Germany, UK and Sweden, centres from these countries were treated as individual study locations (Potsdam, Heidelberg, Oxford, Cambridge, Umeå, Malmö) in all analyses. All participants gave written informed consent, and the study was approved by the local ethics committee in the participating countries and the Internal Review Board of the International Agency for Research on Cancer.

Standard anthropometric data and biological samples were collected from 346,055 of 455,680 individuals from the eight cohorts. Individuals with incident diabetes (*n* = 5,821) at baseline were excluded.

#### Case ascertainment

We used a pragmatic, high-sensitivity approach for case ascertainment, including a review of the existing EPIC datasets at each centre via various sources of evidence, such as self-report, linkage to primary care registers, secondary care registers, medication registers, hospital admissions and mortality data. Cases in Denmark and Sweden were not ascertained by self-report, but identified via local and national diabetes and pharmaceutical registers, and hence treated as verified [14]. Follow-up was censored on 31 December 2007 or the date of death, whichever occurred earlier. In total, 12,403 verified incident type 2 diabetes cases were identified [14].

The date of diagnosis for incident cases was set as either of the following: the date of diagnosis reported by the doctor, the earliest date that diabetes was recorded in medical records, the date of inclusion in the diabetes registry, the date reported by the participant or the date of the questionnaire in which diabetes was first reported. If the date of diagnoses could not be ascertained from any of the sources listed above, the midpoint between recruitment and censoring was used [14].

#### Subcohort

A subcohort of 16,835 individuals was randomly selected from those for whom stored blood and buffy coat were available, stratified by centre. To account for the later exclusion of individuals with prevalent diabetes from InterAct analyses, we sampled more individuals in the subcohort than would be expected from the proportion of prevalent type 2 diabetes cases in each centre. After exclusion of 548 individuals with prevalent diabetes, 129 without information on reported diabetes status, four with post-censoring diabetes and 220 individuals with missing data on physical activity, a total of 15,934 subcohort individuals (6,009 men and 9,925 women) were included in the analysis. Due to the random selection, this subcohort also included a random set of 778 individuals who developed incident type 2 diabetes during follow-up [14].

### Anthropometric measurements

Body weight (kilograms) and height (centimetres) were measured according to standardised procedures without shoes [15], except for the centres at Oxford (UK) and France, where self-reported anthropometric values at baseline were used. Waist circumference (centimetres) was measured at the narrowest torso circumference or at the midpoint between the lower ribs and iliac crest, except for one study centre (Umeå, Sweden), where data on WC were not available. Weight and waist measurements were corrected to account for protocol differences between centres as previously described [15]. BMI was calculated as body weight (kilograms) divided by height squared (square metres). Individuals were categorised into normal-weight (BMI <25 kg/m^2^), overweight (BMI 25–30 kg/m^2^) and obese (BMI ≥30 kg/m^2^) groups. We categorised participants as abdominally lean or obese based on the International Diabetes Federation [16] sex-specific cut points for WC (women ≥80 cm, men ≥94 cm) in whites.

### Physical activity

Physical activity was assessed at baseline by a validated self-report questionnaire [17] combining occupational physical activity and leisure time physical activity (LTPA). Briefly, overall physical activity was assessed from three questions referring to the past year and an index was derived by allocating individuals to four categories of overall activity (inactive, moderately inactive, moderately active and active) [17]. We also derived an index for LTPA only, by excluding the occupational component from the physical activity index.

In one of the centres (Umeå, Sweden) a slightly different questionnaire was used to assess physical activity. From this questionnaire, we derived a four-category index that was similar to that derived from all other study locations, but based on two questions on occupational physical activity and LTPA, respectively [17].

#### Physical activity calibration sub-study

We examined the validity of the four-category physical activity index in 1,941 participants of similar age and sex to those in the original EPIC cohort [17], using combined movement and heart rate-sensing as our criteria [18, 19]. Physical activity energy expenditure (PAEE) increased significantly by increasing categories of self-reported physical activity (*p* < 0.0001 for trend), with a significant correlation between measured PAEE and the categorical physical activity index being observed in all countries (Spearman correlation 0.17 to 0.37, *p* < 0.01). Further calibration results suggest that the average PAEE across categories of physical activity were: inactive (36 kJ/kg daily); moderately inactive (41 kJ/kg daily); moderately active (46 kJ/kg daily); active (51 kJ/kg daily).

### Assessment of covariates

Dietary intakes at baseline were measured using country-specific validated food frequency questionnaires and expressed as total daily energy intake [20].

Standard questionnaires were used at baseline to collect information on the participants’ socio-demographic characteristics, smoking status (never smoker, former smoker, current smoker), educational level (none, primary school, technical school, secondary school, university degree) and alcohol consumption (grams/day).

### Statistics

All analyses were performed separately in men and women. Means and SDs for continuous variables, numbers and percentages for categorical variables, and incidence rates of diabetes were calculated across the four physical activity categories. Hazard ratios per category difference in physical activity were estimated separately with data from each location, using Prentice-weighted Cox regression, with age as the underlying timescale and additionally adjusting for educational level, smoking status, alcohol consumption and energy intake. These HRs were then combined across locations using random effects meta-analysis, and *I* ^2^, the percentage of variation in the HRs that is due to heterogeneity between locations, was calculated. The analysis was repeated using LTPA as the exposure. Individuals were also categorised according to levels of (1) BMI (three groups) and physical activity (four groups), and (2) WC (two groups) and physical activity (four groups), and HRs estimated using Prentice-weighted Cox regression, with active as the reference group across strata for BMI and WC. Because in some locations there were very few individuals in some of the groups defined by (1) and (2) above, these latter models were fitted in the full dataset, with adjustment for location. Analyses were performed using Stata version 11.1. (Stata, College Station, TX, USA).

## Results

The present report includes 11,669 men and 15,695 women, of whom 5,660 and 5,570, respectively, were incident cases of type 2 diabetes. Based on subcohort data, 6.3% of men and 3.9% of women developed type 2 diabetes over a median follow-up time of 12.3 years (192,876 total person-years).

**Fig. 1:**
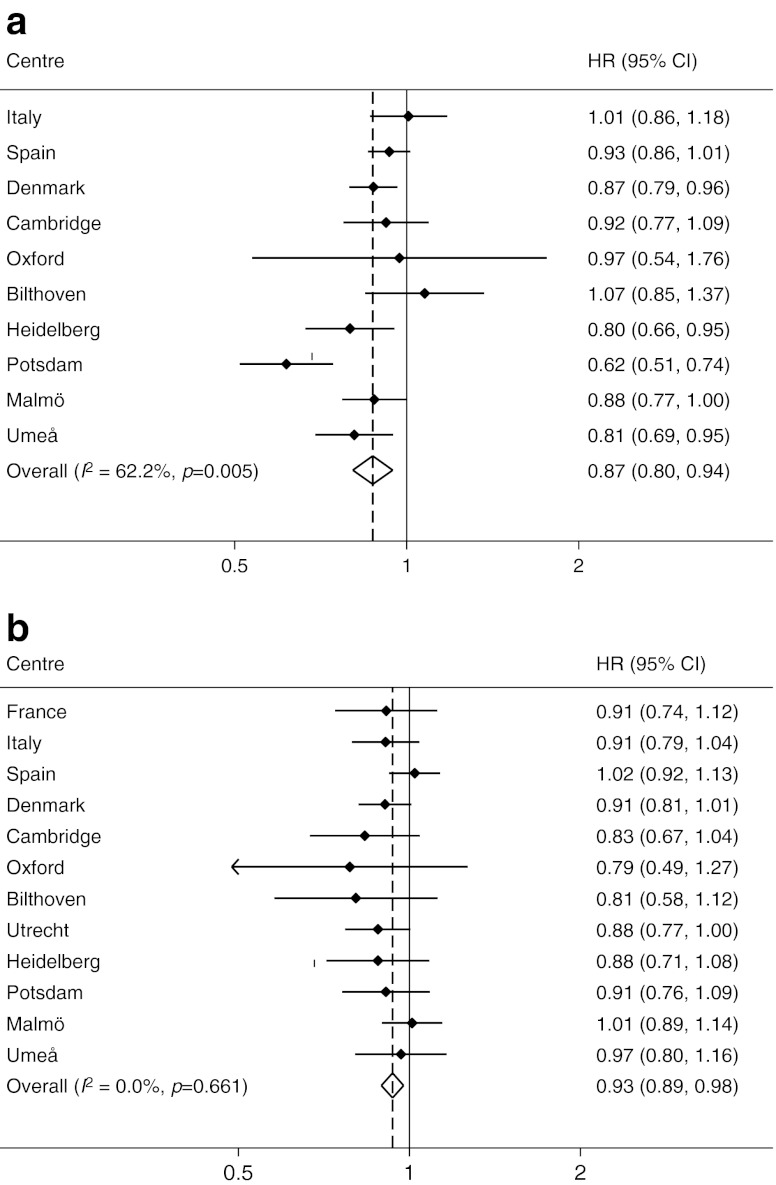
HR (95% CIs) of incident diabetes per one-level difference in physical activity in (**a**) men and (**b**) women. Models are adjusted for baseline BMI, education, smoking status, alcohol consumption and energy intake

**Fig. 2:**
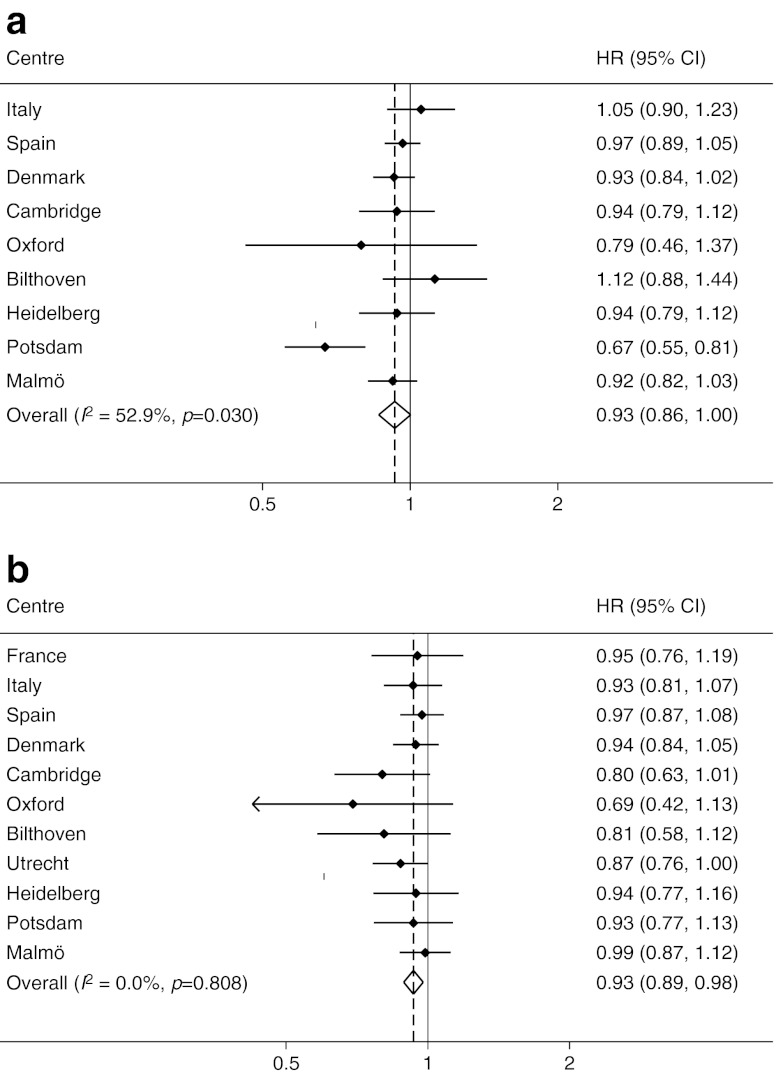
HR (95% CIs) of incident diabetes per one-level difference in physical activity (**a**) men and (**b**) women. Models are adjusted for baseline WC, education, smoking status, alcohol consumption and energy intake

Table 1 describes the characteristics of randomly selected subcohort participants stratified by sex and categories of physical activity. Figure 1 shows the estimated HRs and 95% CIs of incident diabetes per one category difference in physical activity in men (Fig. 1a) and women (Fig. 1b). After adjustment for study centre, education, smoking status, alcohol consumption, energy intake and BMI, a one level difference in physical activity (e.g. between inactive and moderately inactive) was associated with a 13% relative reduction in risk of incident type 2 diabetes in men and a 7% risk reduction in women (Fig. 1a, b). We thereafter substituted BMI by WC as a confounding variable; the effects were then slightly attenuated in men (HR 0.93, 95% CI 0.86, 1.00), but unchanged in women (HR 0.93, 95% CI 0.89, 0.98) (Fig. 2a, b).

**Table 1:** Characteristics of participants included in the random subcohort, stratified according to baseline overall physical activity

Characteristic	Activity category			
	Inactive	Moderately inactive	Moderately active	Active
Men				
*n*	1,125	1,854	1,533	1,497
Diabetes incidence^a^	5.78	5.93	5.36	4.14
Age (years)	55.4 ± 9.2	53.2 ± 8.6	52.2 ± 8.6	51.43 ± 8.8
Height (cm)	173.6 ± 7.5	174.4 ± 7.2	174.1 ± 7.5	173.80 ± 7.50
Weight (kg)	81.5 ± 12.2	80.9 ± 11.5	80.4 ± 11.8	80.14 ± 11.6
BMI (kg/m^2^)	27.0 ± 3.7	26.6 ± 3.5	26.5 ± 3.7	26.54 ± 3.53
Waist (cm)	97.6 ± 10.6	95.4 ± 10.0	94.6 ± 9.8	93.82 ± 9.8
Energy intake (kJ/day)	9,790 ± 2,642	10,202 ± 2,643	10,382 ± 2,642	10,957 ± 2,870
Alcohol (g/day)	18.1 ± 22.6	22.3 ± 24.0	22.4 ± 23.8	23.1 ± 24.0
School level, *n* (%)				
None (*n* = 334)	61 (5.51)	95 (5.17)	92 (6.03)	86 (5.80)
Primary (*n* = 2,039)	390 (35.23)	552 (30.07)	519 (34.03)	578 (38.95)
Technical/professional (*n* = 1,350)	231 (20.87)	385 (20.97)	347 (22.75)	387 (26.08)
Other secondary (*n* = 788)	151 (13.64)	261 (14.22)	217 (14.23)	159 (10.71)
Higher (*n* = 1,441)	274 (24.75)	543 (29.58)	350 (22.95)	274 (18.46)
Smoking status, *n* (%)				
Never (*n* = 1,896)	315 (28.05)	593 (32.12)	502 (33.00)	486 (32.57)
Ex (*n* = 2,195)	397 (35.35)	692 (37.49)	548 (36.03)	558 (37.40)
Current (*n* = 1,891)	411 (36.60)	561 (30.39)	471 (30.97)	448 (30.03)
Hypertension, *n* (%)				
No (*n* = 4,664)	844 (77.01)	1,418 (78.65)	1,217 (81.68)	1,185 (83.80)
Yes (*n* = 1,139)	255 (22.99)	385 (21.35)	273 (18.32)	229 (16.20)
Hyperlipidaemia, *n* (%)				
No (*n* = 3,198)	572 (77.09)	973 (73.38)	828 (75.62)	825 (76.96)
Yes (*n* = 1,037)	170 (22.91)	353 (26.62)	267 (24.38)	247 (23.04)
Myocardial infarction, *n* (%)				
No (*n* = 5,716)	1,025 (96.06)	1,756 (97.45)	1,487 (97.89)	1,448 (97.64)
Yes (*n* = 155)	42 (3.94)	46 (2.55)	32 (2.11)	35 (2.36)
Stroke, *n* (%)				
No (*n* = 5,327)	899 (97.29)	1,684 (99.29)	1,358 (98.98)	1,386 (98.86)
Yes (*n* = 67)	25 (2.71)	12 (0.71)	14 (1.02)	16 (1.14)
Women				
*n*	2,676	3,487	2,069	1,693
Diabetes incidence^a^	4.57	3.03	2.57	2.54
Age (years)	52.6 ± 9.7	52.4 ± 9.3	51.5 ± 8.8	51.7 ± 8.9
Height (cm)	159.1 ± 6.7	161.4 ± 6.5	162.9 ± 6.4	163.4 ± 6.4
Weight (kg)	68.2 ± 12.4	66.4 ± 11.5	65.9 ± 11.3	66.3 ± 10.9
BMI (kg/m^2^)	27.0 ± 4.9	25.5 ± 4.4	24.9 ± 4.1	24.8 ± 4.0
Waist (cm)	84.7 ± 11.7	80.9 ± 11.2	78.9 ± 10.4	79.3 ± 10.2
Energy intake (kJ/day)	8,023 ± 2,315	8,068 ± 2,150	8,138 ± 2,170	8,188 ± 2,191
Alcohol (g/day)	5.90 ± 10.10	8.0 ± 11.7	8.7 ± 12.0	9.4 ± 12.4
School level, *n* (%)				
None (*n* = 875)	488 (18.53)	275 (8.00)	79 (3.88)	33 (1.97)
Primary (*n* = 3,213)	1,115 (42.35)	1,082 (31.47)	550 (26.99)	466 (27.80)
Technical/professional (*n* = 2,286)	384 (14.58)	841 (24.46)	532 (26.10)	529 (31.56)
Other secondary (*n* = 1,614)	319 (12.12)	589 (17.13)	392 (19.23)	314(18.74)
Higher (*n* = 2,375)	327 (12.42)	651 (18.94)	485 (23.80)	334 (19.93)
Smoking status, *n* (%)				
Never (*n* = 5,532)	1,655 (62.10)	1,941 (56.02)	1,081 (52.60)	855 (50.68)
Ex (*n* = 2,111)	406 (15.23)	748 (21.59)	500 (24.33)	457 (27.09)
Current (*n* = 2,229)	604 (22.66)	776 (22.40)	474 (23.07)	375 (22.23)
Hypertension, *n* (%)				
No (*n* = 7,993)	2,097 (78.95)	2,801 (81.38)	1,717 (84.37)	1,378 (83.21)
Yes (*n* = 1,796)	559 (21.05)	641 (18.62)	318 (15.63)	278 (16.79)
Hyperlipidaemia, *n* (%)				
No (*n* = 6,539)	1,859 (83.25)	2,252 (83.13)	1,328 (87.25)	1,100 (88.71)
Yes (*n* = 1,165)	374 (16.75)	457 (16.87)	194 (12.75)	140 (11.29)
MI, *n* (%)				
No (*n* = 9,731)	2,594 (98.97)	3,404 (99.36)	2,050 (99.56)	1,683 (99.76)
Yes (*n* = 62)	27 (1.03)	22 (0.64)	9 (0.44)	4 (0.24)
Stroke, *n* (%)				
No (*n* = 9,192)	2,458 (99.15)	3,262 (99.39)	1,896 (99.37)	1,576 (99.18)
Yes (*n* = 66)	21 (0.85)	20 (0.61)	12 (0.63)	13 (0.82)

Values are mean ± SD, unless specified otherwise

^a^Per 1,000 person-years

The significant heterogeneity by study location that was observed in men for the associations including BMI (overall *I* ^2^ 62.2%, *p* = 0.005) and WC (overall *I* ^2^ 52.9%, *p* = 0.030) was partly explained by the low proportion of men in the obese category in some of the study locations.

We then examined whether lower levels of LTPA increased the risk of incident type 2 diabetes in similar models, as above. In men, the HR for a one category difference in LTPA was 0.90 (95% CI 0.82, 0.99) following adjustment for BMI and the confounders listed above, and 0.94 (95% CI 0.88, 1.03) when analyses were adjusted for WC rather than BMI. In women, the protective effect of LTPA was evident following adjustment for BMI or WC, with the same effect sizes for general and central obesity (HR 0.90, 95% CI 0.85, 0.95).

We next examined the combined effects of physical activity and BMI on incident diabetes stratified by sex across BMI categories (Table 2). The increased risk of incident type 2 diabetes associated with lower levels of physical activity was evident across BMI strata in both sexes, with the exception of obese women. The results for the stratified association analyses of physical activity and the dichotomous variable for WC are shown in Table 3. The risk of developing diabetes increased by lower levels of physical activity in abdominally lean and abdominally obese men and women. However, the increased risk associated with low levels of physical activity was more pronounced in abdominally lean than in abdominally obese women, whereas in men the increased risk with lower levels of physical activity was similar regardless of abdominal obesity.

**Table 2:** Combined effects (HR [95% CI]) of overall physical activity and BMI on incident diabetes in men (*n* = 11,669) and women (*n* = 15,695)

Variable	Activity category			
	Active	Moderately active	Moderately inactive	Inactive
Men				
*n*	2,645	2,794	3,660	2,600
Normal weight (BMI <25 kg/m^2^)	1.00	0.83 (0.62, 1.10)	1.32 (1.01, 1.72)	1.81 (1.34, 2.43)
Overweight (BMI 25–30 kg/m^2^)	1.00	1.09 (0.93, 1.28)	1.15 (0.99, 1.34)	1.36 (1.14, 1.62)
Obese (BMI >30 kg/m^2^)	1.00	1.11 (0.87, 1.40)	1.36 (1.08, 1.71)	1.38 (1.08, 1.78)
Women				
*n*	2,458	3,134	5,428	4,675
Normal weight (BMI <25 kg/m^2^)	1.00	1.12 (0.88, 1.41)	1.13 (0.90, 1.40)	1.50 (1.17, 1.93)
Overweight (BMI 25–30 kg/m^2^)	1.00	1.17 (0.96, 1.43)	1.17 (0.97, 1.41)	1.41 (1.15, 1.72)
Obese (BMI >30 kg/m^2^)	1.00	1.19 (0.91, 1.55)	1.17 (0.92, 1.48)	1.2 (0.94, 1.54)

Values are HR (95% CI), unless specified otherwise

Models are adjusted for study location, education (none, primary, technical/other secondary, professional), smoking status (never, former, current), alcohol consumption (grams/day) and energy intake (kilojoules/day)

*p* for linear trend across categories of BMI in men were: BMI <25 kg/m^2^, *p* < 0.001; BMI 25–30 kg/m^2^, *p* < 0.001; BMI >30 kg/m^2^, *p* = 0.003; corresponding *p* values in women: BMI <25 kg/m^2^, *p* = 0.003; BMI 25–30 kg/m^2^, *p* = 0.00; BMI >30 kg/m^2^, *p* = 0.25

*p* for interaction (physical activity × BMI): *p* = 0.008 (in women)

**Table 3:** Combined effects of overall physical activity and central obesity (WC) on incident diabetes in men (*n* = 10,745) and women (*n* = 14,817)

Variable	Activity category			
	Active	Moderately active	Moderately inactive	Inactive
Men				
*n*	2,511	2,524	3,449	2,261
WC (<94 cm)	1.00	1.11 (0.88, 1.40)	1.21 (0.98, 1.51)	1.44 (1.11, 1.87)
WC (≥94 cm)	1.00	1.05 (0.90, 1.22)	1.2 (1.09, 1.46)	1.38 (1.17, 1.62)
Women				
*n*	2,294	2,890	5,204	4,429
WC (<80 cm)	1.00	1.06 (0.82, 1.39)	1.22 (0.96, 1.56)	1.57 (1.19, 2.07)
WC (≥80 cm)	1.00	1.15 (0.98, 1.36)	1.08 (0.92, 1.25)	1.19 (1.01, 1.39)

Values are HR (95% CI), unless specified otherwise

Models are adjusted for study location, education (none, primary, technical/other secondary, professional), smoking status (never, former, current), alcohol consumption (grams/day), energy intake (kilojoules/day) and BMI

*p* for linear trend across categories for WC in men were: WC <94 cm, *p* = 0.009; WC ≥94 cm, *p* < 0.001; corresponding *p* values in women: WC <80 cm, *p* = 0.002; WC ≥80, *p* < 0.001

Overall, in combined association analyses (using normal-weight and physically active as the reference group), the effect of BMI on the risk of diabetes appeared to be more pronounced than the effect of lower levels of physical activity (Electronic supplementary material [ESM] Tables 1 and 2).

In sensitivity analyses, we excluded all participants (*n* = 10,287) who were diagnosed with stroke, heart attack, hypertension and hyperlipidaemia at baseline; the conclusions were unchanged (data not shown).

## Discussion

Higher levels of physical activity were associated with a reduced risk of developing type 2 diabetes independently of general adiposity in men, and independently of general and abdominal adiposity in women. The protective effect of physical activity was evident in normal-weight, overweight and obese individuals (except for obese women), and in lean and abdominally obese men and women. However, the protective effect of physical activity appeared to be more pronounced in abdominally obese men than women.

While previous studies [3–8] have examined the protective effect of LTPA or regular structured exercise on incident type 2 diabetes, our measure of physical activity also included occupational physical activity, which is a major component of daily life. In analyses considering LTPA, the magnitude of association with incident diabetes was weaker, although still statistically significant in men, whereas in women the protective effect of LTPA was more pronounced than with overall physical activity. This suggests that total physical activity, and not only LTPA, is important for reducing the risk of diabetes, at least in men. However, the difference in magnitude of association between men and women when comparing LTPA with overall physical activity may also be due to a greater relative contribution from LTPA to the combined overall score in women. This may be because men were more likely to engage in heavy manual labour than women when the baseline data were collected in the EPIC Study in the 1990s. Future studies including a more detailed characterisation of domain-specific types of physical activity, ideally combined with objective monitoring of physical activity, are needed to elucidate the domain-specific contribution of physical activity towards reducing the risk of developing type 2 diabetes.

Few previous studies have examined the combined effects of physical activity, and of general and abdominal adiposity on incident diabetes. In those that have addressed this question, strong conclusions have been limited by the use of homogenous samples of men [8] or women [7, 10], the small number of cases [9], and simple dichotomisation of physical activity into inactive and active groups [7, 8], or of obesity into obese and non-obese groups [9]. Weinstein et al [7] observed that physical activity and BMI were associated with increased risk of developing type 2 diabetes in women (1,361 cases). However, when comparing active versus inactive women across BMI groups in a combined analysis, no protective effect of physical activity on the risk of developing diabetes was observed [7]. In line with our observations, Rana et al [10] observed a protective effect of leisure time exercise across BMI categories using data from the Nurse’s Health Study (4,030 cases). Siegel et al [8] analysed data from the Physicians’ Health Study (1,836 cases) and found that physically inactive men who were of normal weight or overweight had an increased risk of developing type 2 diabetes compared with their active counterparts, although no protective effect of physical activity was observed in obese men. Our results comprise a much greater number of incident type 2 diabetes cases (12,403) in men and women, and go beyond previous findings in that a higher level of physical activity was associated with a decreased risk of type 2 diabetes across all BMI groups.

One previous study in women [10] suggested that physical activity did not provide any protective effect across tertiles of abdominal obesity. Our data, which include about three times more cases, contradict this conclusion, as we observed a graded dose–response association in abdominally lean and obese men and women. The difference in findings may be explained by our larger and more heterogeneous sample of participants, and possibly by differences in how physical activity was assessed.

As in previous studies [7–10], we observed that obesity may be a stronger determinant of incident type 2 diabetes than physical activity. Even among active men and women, overweight and obesity were associated with a substantially increased risk of diabetes in a dose–response manner (ESM Tables 1 and 2). However, caution is warranted when directly comparing the relative magnitude of associations between exposures measured with different degrees of error. Physical activity was assessed by self-report, which is less precise as a measure of usual activity than the measurement of BMI and WC as indicators of usual adiposity. Measurement error in the exposure variable is inevitable, but is likely to be non-differential and would bias the association between physical activity and the risk of developing diabetes towards the null. The level of measurement error in self-reported physical activity may also differ according to obesity status, as obese individuals may over-report their levels of activity. This may partly explain why the protective effect of physical activity was less pronounced in obese groups and especially in obese women.

Our study has some additional potential limitations. Case ascertainment and verification status differed between study locations and screening for blood glucose was not feasible. However, we identified 12,403 incident cases of type 2 diabetes that were verified through multiple sources in each study location. Even if some under-diagnosis may have occurred, the effect on the observed measure of association would be conservative. We only assessed physical activity and anthropometry at baseline, and any change in physical activity and/or obesity between baseline and follow-up might have caused misclassification, therefore attenuating the associations observed. Although we adjusted our analyses for several potential lifestyle-related variables, including dietary intake, alcohol consumption and smoking, residual confounding may have persisted.

The association between physical activity and type 2 diabetes independently of obesity is biologically plausible [21, 22]. However, higher levels of physical activity may not completely abolish the counteracting and detrimental effects of obesity on diabetes risk [23].

Our results have some potential implications for public health. Results from our calibration study suggest that each increment in physical activity corresponded to approximately 380 and 460 kJ PAEE per day in men and women, respectively. Thus the 19 to 81 % risk reduction observed between extreme categories of physical activity across BMI and abdominal obesity groups could correspond to about 1,200 kJ/day of PAEE. Although these estimates should be interpreted with caution, they were derived from an independent sample of almost 2,000 men and women from all ten EPIC countries, who were measured twice by individually calibrated heart rate- and movement-sensing [17].

Previous lifestyle interventions have been successful in reducing the incidence of type 2 diabetes in those at high risk of developing the disease [24, 25]. However, those studies were not designed to examine the amount of physical activity needed to reduce the risk of diabetes and whether physical activity was beneficial independently of body weight reduction. Future large-scale randomised controlled trials including a detailed characterisation of physical activity and body composition are needed to address this question.

In conclusion, physical activity predicts the development of type 2 diabetes independently of general and abdominal adiposity. Higher levels of physical activity are associated with a substantial risk reduction across BMI categories, and in abdominally lean and obese men and women. The promotion of physical activity appears to have beneficial effects on the prevention of type 2 diabetes, regardless of the degree of overall and abdominal adiposity.

### Electronic supplementary material

Below is the link to the electronic supplementary material.

ESM 1 (PDF 30 kb)

ESM 2 (PDF 29 kb)

## Appendix

### The InterAct Consortium list of authors is as follows:

U. Ekelund (MRC Epidemiology Unit, Cambridge, UK), L. Palla (MRC Epidemiology Unit, Cambridge, UK), S. Brage (MRC Epidemiology Unit, Cambridge), PW. Franks (Umeå University, Umeå, Sweden and Lund University, Malmö, Sweden), T. Peters (MRC Epidemiology Unit, Cambridge, UK), B. Balkau (INSERM, University Paris Sud, Villejuif, France), MJ. T. Diaz (Department of Epidemiology, Murcia Regional Health Council, Murcia, Spain), JM. Huerta (Department of Epidemiology, Murcia Regional Health Council, Murcia, Spain), C. Agnoli (Nutritional Epidemiology Unit, Istituto Nazionale dei Tumori, Milan, Italy), L. Arriola (Public Health Division of Gipuzkoa, San Sebastian, Spain), E. Ardanaz (Navarre Public Health Institute, Pamplona, Spain), H. Boeing (German Institute of Human Nutrition Potsdam-Rehbruecke, Nuthetal, Germany), F. Clavel-Chapelon (INSERM, University Paris Sud, Villejuif, France), F. Crowe (University of Oxford, Oxford, UK), G. Fagherazzi (INSERM, University Paris Sud, Villejuif, France), L. Groop (Lund University, Malmö, Sweden), P. Hainaut (IARC, Lyon, France), N. Føns Johnsen (Danish Cancer Society, Copenhagen, Denmark), R. Kaaks (German Cancer Research Centre, Heidelberg, Germany), KT. Khaw (University of Cambridge, Cambridge, UK), TJ. Key (University of Oxford, Oxford, UK), B. de Lauzon-Guillain (INSERM, University Paris Sud, Villejuif, France), A. May (University Medical Centre Utrecht, Utrecht, the Netherlands), E. Monninkhof (University Medical Centre Utrecht, Utrecht, the Netherlands), C. Navarro (Department of Epidemiology, Murcia Regional Health Council, Murcia, Spain), P. Nilsson (Lund University, Malmö, Sweden), J. Nautrup Østergaard (Aarhus University Hospital, Aalborg, Denmark), T. Norat (Imperial College London, UK), K. Overvad (Aarhus University Hospital, Aalborg, Denmark), D. Palli (Cancer Research and Prevention Institute, Florence, Italy), S. Panico (Federico Ii University Naples, Italy), ML. Redondo (Public Health Directorate, Asturias, Spain), F. Ricceri (Human Genetics Foundation, Turin, Italy), O. Rolandsson (Umeå University, Umeå, Sweden), D. Romaguera (Imperial College London, UK), I. Romieu (IARC, Lyon, France), MJ. Sánchez Pérez (Andalusian School of Public Health, Granada Spain), N. Slimani (IARC, Lyon, France), A. Spijkerman (RIVM, Bilthoven, the Netherlands), B. Teucher (German Cancer Research Centre, Heidelberg, Germany), A. Tjonneland (Danish Cancer Society, Copenhagen, Denmark), N. Travier (Catalan Institute of Oncology, Barcelona, Spain), R. Tumino (Cancer Registry and Histopathology Unit, Ragusa, Italy), W. Vos (RIVM, Bilthoven, the Netherlands), M. Vigl (German Institute of Human Nutrition Potsdam-Rehbruecke, Nuthetal, Germany), S. Sharp (MRC Epidemiology Unit, Cambridge, UK), C. Langenberg (MRC Epidemiology Unit, Cambridge, UK), N. Forouhi (MRC Epidemiology Unit, Cambridge, UK), E. Riboli (Imperial College London, UK), E. Feskens (University of Wageningen, Wageningen, the Netherlands), NJ. Wareham (MRC Epidemiology Unit, Cambridge, UK).

## Abbreviations

EPIC: European Prospective Investigation into Cancer and Nutrition
LTPA: Leisure time physical activity
PAEE: Physical activity energy expenditure
WC: Waist circumference
