# Supplementary material for: Physical activity reduces the risk of incident type 2 diabetes in general and in abdominally lean and obese men and women: the EPIC–InterAct Study
Source: Diabetologia. 2012 Apr 21;55(7):1944–52. doi: 10.1007/s00125-012-2532-2 (PMC3369127; doi:10.1007/s00125-012-2532-2)
Supplement: Supplementary file 1 — (PDF 30 kb) [file 125_2012_2532_MOESM1_ESM.pdf]

**ELECTRONIC SUPPLEMENTARY MATERIAL**

**Supplementary Table 1.** Combined effects (Hazard Ratios; 95% CI) of overall physical activity and BMI on incident diabetes in men and women (Normal-weight and Active as the reference group).

| | Active (n=2,645) | Moderately Active (n=2,794) | Moderately Inactive (n=3,660) | Inactive (n=2,600) |
|---|---|---|---|---|
| **Men** | | | | |
| Normal weight (BMI<25 kg/$m^2$) | 1.00 | 0.80 (0.61-1.06) | 1.20 (0.94-1.55) | 1.69 (1.28-2.22) |
| Over-weight (BMI 25-30 kg/$m^2$) | 1.58 (1.23-2.02) | 1.77 (1.38-2.27) | 1.86 (1.46-2.37) | 2.27 (1.76-2.93) |
| Obese (BMI>30 kg/$m^2$) | 1.73 (1.19-2.52) | 1.91 (1.32-2.76) | 2.52 (1.77-3.60) | 2.37 (1.65-3.40) |

| | Active (n=2,458) | Moderately Active (n=3,134) | Moderately Inactive (n=5,428) | Inactive (n=4,675) |
|---|---|---|---|---|
| **Women** | | | | |
| Normal weight (BMI<25 kg/$m^2$) | 1.00 | 1.04 (0.83-1.30) | 1.08 (0.87-1.33) | 1.43 (1.14-1.80) |
| Over-weight (BMI 25-30 kg/$m^2$) | 2.15 (1.69-2.73) | 2.55 (2.02-3.23) | 2.49 (2.00-3.10) | 2.90 (2.30-3.64) |
| Obese (BMI>30 kg/$m^2$) | 3.57 (2.54-5.03) | 4.16 (2.96-5.85) | 3.97 (2.89-5.45) | 4.21 (3.09-5.74) |

Models are adjusted for study location education (no/primary/technical/other secondary, professional), smoking status (never/former/current), alcohol consumption (g/day), energy intake (kcal/day) and baseline BMI.
