# Supplementary material for: Physical activity reduces the risk of incident type 2 diabetes in general and in abdominally lean and obese men and women: the EPIC–InterAct Study
Source: Diabetologia. 2012 Apr 21;55(7):1944–52. doi: 10.1007/s00125-012-2532-2 (PMC3369127; doi:10.1007/s00125-012-2532-2)
Supplement: Supplementary file 2 — (PDF 29 kb) [file 125_2012_2532_MOESM2_ESM.pdf]

Supplementary Table 2. Joint effects (Hazard Ratios; 95% CI) of overall physical activity and central obesity (waist circumference) with incident diabetes in men and women (Abdominally lean and Active as the reference group).

	Active (n=2,511)	Moderately Active (n=2,524)	Moderately Inactive (n=3,449)	Inactive (n=2,261)
**Men**				
Waist circumference (<94 cm)	1.00	1.09 (0.88-1.36)	1.19 (0.97-1.47)	1.45 (1.14-1.85)
Waist circumference (≥94 cm)	1.44 (1.16-1.80)	1.54 (1.24-1.92)	1.77 (1.43-2.19)	1.85 (1.48-2.33)

	Active (n=2,294)	Moderately Active (n=2,890)	Moderately Inactive (n=5,204)	Inactive (n=4,429)
**Women**				
Waist circumference (<80 cm)	1.00	0.98 (0.76-1.26)	1.13 (0.90-1.42)	1.36 (1.06-1.75)
Waist circumference (≥80 cm)	1.95 (1.51-2.50)	2.28 (1.78-2.91)	2.10 (1.67-2.65)	2.35 (1.84-2.99)

Models are adjusted for study centre, education (no/primary/technical/other secondary, professional), smoking status (never/former/current), alcohol consumption (g/day) energy intake (kcal/day) and baseline waist circumference.
